# Prevalence and antimicrobial susceptibility of *Salmonella* and *Shigella* species isolated from diarrheic children in Ambo town

**DOI:** 10.1186/s12887-020-1970-0

**Published:** 2020-02-27

**Authors:** Wagi Tosisa, Adane Mihret, Asnake Ararsa, Tadesse Eguale, Tamrat Abebe

**Affiliations:** 1 Department of Medical Laboratory Sciences, College of Medical and Health Sciences, Ambo University, P. O. Box 19, Ambo, Ethiopia; 2 Armauer Hansen Research Institute, Addis Ababa, Ethiopia; 3 Deakin University, School of Exercise and Nutrition Sciences, Burwood, Australia; 4 Aklilu Lemma Institute of Pathobiology, Addis Ababa University, P.O. Box 1176, Addis Ababa, Ethiopia

**Keywords:** *Salmonella*, *Shigella*, Antibiotic resistance, Childhood diarrhea, Ambo, Ethiopia

## Abstract

**Background:**

Diarrhea, particularly of enteric bacterial pathogen, remains a major cause of morbidity and mortality in Ethiopia. Despite the high prevalence of diarrheal disease among under-five children, antibiotic resistance of bacterial pathogens test is not part of routine childcare in the study area. This study aimed to investigate the prevalence and antimicrobial susceptibility status of *Salmonella* and *Shigella* species among diarrheic children attending public health institutions in Ambo town, west Showa, Ethiopia.

**Methods:**

Institutional based, cross-sectional study was carried out from January to July 2014 among 239 diarrheic children below five years of age in Ambo town, Ethiopia. Information about patient demographics, signs, and symptoms was obtained from the parents/guardians of each child using a questionnaire. Stool samples from diarrheic children were collected and processed for isolation of *Salmonella* and *Shigella* using conventional microbiology procedures. Suspected *Salmonella* isolates were confirmed by genus-specific PCR and serotyped using a slide agglutination test. Susceptibility to 10 commonly used antimicrobials was assessed using the Kirby Bauer disc diffusion method.

**Results:**

From the 239 children screened, only nine (3.8%) of them were positive for either *Salmonella* (*n* = 3) or *Shigella* (*n* = 6) and 19 (7.9%) positive for the intestinal parasite. Three species of *Shigella* were identified: *Shigella flexinari* (n = 3), *Shigella boydii* (*n* = 2), and *Shigella sonnei* (*n* = 1). The three *Salmonella* isolates were S. chicago, S. caracas, and S. saintpaul. Salmonella and *Shigella* isolates were resistant to ampicillin (88.9%), followed by tetracycline (66.7%), cotrimoxazole (55.6%), chloramphenicol (44.4%), amoxicillin (33.3%), nalidixic acid (11.1%) and cefotaxime (11.1%). All isolates were sensitive to amikacin, ciprofloxacin, and gentamycin.

**Conclusion:**

In this study, either *Salmonella* or *Shigella* species were detected only in 3.8% of diarrheic children in Ambo town, suggesting the dominance of other causes of diarrhea in the study area. A further study targeting other causes of diarrhea should be conducted to establish the major causes of childhood diarrhea in the study area.

## Background

Infectious diarrhea, especially those due to enteric bacterial pathogens, remains a significant public health problem worldwide. While it primarily contributes to morbidity in developed countries, it accounts for significant mortality among children in low and middle-income countries (LMICs) [1]. Despite declining diarrhea-related death in the last 20 years [2], it still accounts for 21% of under-five child mortality, which translates to 2.5 million child deaths [3]. Africa and South Asia are still home to more than 80% of child deaths. Among 15 high burden countries that have three-quarters of all deaths from diarrhea, Ethiopia was ranked fifth [4], by having 27% diarrhea-related deaths [5].

The increasing antimicrobial resistance among enteric pathogens has become a contemporary global health threat. Notably, the *Shigella, Vibrio cholerae, Enteropathogenic E. coli (EPEC)*, and *Salmonella* species are a critical concern of the developing world responsible for the high rate of diarrhea-related deaths. One reason for increasing antimicrobial resistance is the unrestricted use of over the counter drugs without medical supervision [6].

In Ethiopia, according to a study conducted in Jimma Health Center, 49.6% were positive for an intestinal parasite, *Shigella,* and *Salmonella* species. While *Shigella* species showed 100% resistance to ampicillin, amoxicillin, and cotrimoxazole, *Salmonella* isolates were resistant to amoxicillin. In contrast, all *Shigella* and *Salmonella* species were susceptible to ceftriaxone, ciprofloxacin, and gentamycin [7].

The magnitude of the *Shigella* species resistant to nalidixic acidis*,* an emerging problem in Ethiopia, ranges from 6.5% [8] to 16.7% [7] in Jimma, Southwestern Ethiopia; at 5.9% in Butajira [9] and 10% in Hawassa [10]. On the contrary*, Shigella* species isolated from diarrheic patients were susceptible to amikacin, ciprofloxacin, and gentamycin in Harar [11] and Jimma [9].

With regard to *Salmonella*, while there was a high level of resistance to ampicillin in Harar (100%) [11], Bahir Dar (93.9%) [12], Addis Ababa (82.3%) [13], Jimma (62.5%) [7], and Butajira (60%) [9], all isolates from Hawassa were susceptible to ampicillin [14]. This level difference might be related to the heavy reliance on empirical antibiotics treatment for infectious diseases in Ethiopia and irregularities in implementing the treatment protocol. Consequently, the problem increases the risk of the emergence of antibiotic-resistant bacteria strains [15].

The level of antibiotic resistance gauges the clinical and the community malpractices related to the use of antibiotics and the associated risk of emerging infections. Despite this, Ethiopia’s health facilities do not routinely perform the test for antibiotic resistance, at least among the most vulnerable segment of the population, the children affected by diarrhea. As a result, there is a poor understanding of antibiotic resistance on the most common etiologies of diarrhea, *Salmonella,* and *Shigella* species in central Ethiopia. Hence, this study intended to explore the magnitude and antimicrobial susceptibility of *Salmonella* and *Shigella* species isolated from diarrheic children in Ambo town.

## Methods

### Study design, area, and period

An institutional-based, cross-sectional study was carried out in Ambo Town Public Health Institutions (ATPHI)—at Ambo General Hospital, Ambo Health Center, and Awaro Health Center—from January to July 2014. These institutes provide health services for Ambo town and the surrounding districts. Ambo is one of the districts in the Western Shewa Zone, Oromia Region of Ethiopia. The 2007 national census of Ethiopia reported total populations for this district to be 108,406, of whom 54,186 were men and 54,220 were women [16].

### Sample size

The sample size for the study was determined using a single population proportion formula. The prevalence and antimicrobial susceptibility of bacterial pathogens isolated from childhood diarrhea in Kenya were 17.7% [17]. It used as a reference and at a 95% level of confidence and a 10% non-response rate, the total of 250 children with diarrheal disease targeted to be included in the study as;

$$ \mathrm{n}=\frac{{\left(\mathrm{Z}\kern0.1cm \upalpha \right)}^2\left(\mathrm{p}\kern0.1cm \mathrm{q}\right)}{{\mathrm{d}}^2} $$

Where: n = sample size.

Zα/2 = level of confidence.

P = diarrhea prevalence from previous study = 17.7%.

q = 1-p.

d = margin of error (0.05).

The responded client size was 239 children less than five years of age presented with diarrhea to pediatric OPDs and Wards’. Diarrheic stool is defined as having loose or watery stools at least three times per day, or more frequently than usual for an individual (as per the WHO definition) [4]. A systematic random sampling method used to draw participants based pattern of previous patient flow. Accordingly, every other child whom the parents/guardians briefed on the aim of the study agreed and signed the consent to participate included in the study. Children who did not take an antibiotic for the current diarrheal attack included in the study.

### Sample collection, handling, and transport

The collected clinical data includes body temperature, demographic data, and medical history. A single diarrheic stool specimen collected after a physical examination. At a hospital and health centers, the stool samples inoculated in Cary-Blair’s transport medium (CA, USA). The samples transported in a cool box to Ambo University Microbiology Laboratory within four hours of collection, and it processed on the same day.

### Microscopic examination

Stool examination performed at health institution laboratories (Ambo General Hospital, Ambo Health Center, and Awaro Health Center). Macroscopic and microscopic examination of the safeguarded specimens (formalin 10%) completed then focuses on formalin-ether sedimentation for intestinal parasites, WBC, and RBC conducted immediately upon sample collection before being inoculated into Cary-Blair’s transport medium.

### Culture and identification

All stool specimens cultured for isolation of *Shigella* and *Salmonella* species. The collected samples inoculated aerobically first in Selenite F broth (HIMEDIA, India) for the enrichment of *Salmonella* and *Shigella* species. Then, the samples inoculated to Xylose lysine desoxycholate agar (XLD) (OXOID, England) and incubated at 35–37 °C for 14–16 h. A loopful of the fecal suspension directly inoculated onto MacConkey agar (SRL, India), and *Salmonella-Shigella* (SRL, India) agar, and incubated at 37 °C for 18–24 h. MacConkey agar used to characterize most enteric bacteria toward their lactose utilization property, XLD (*Shigella*: red colonies, *Salmonella*: red with/without a black center), and SS agar used for the isolation of *Shigella* and *Salmonella* species. The presumptive colonies of each representative isolates, then characterized using standard biochemical tests.

Biochemical tests performed to characterize the enteric gram-negative bacteria include gram stain morphology, pigment production, motility, urease, citrate, hydrogen sulfide utilization, oxidase, indole, lysine, and sugar fermentation. The media used were nutrient broth (CONDA, Spain), lysine iron agar (LIA) (OXIOD, England), MRVP, Simmons citrate agar (HIMEDIA, India), Kligler iron agar (KIA) (SRL, India), Sulfide-Indole-Motility (SIM), urea broth base (OXIOD, England), Motility Indole Ornithine Medium (MIO) (OXIOD, England). The 3% H_2_O_2_ was used to identify *Salmonella, Shigella* species, and other enteric bacteria as adopted from the Basic laboratory Procedures in Clinical bacteriology WHO (Vandepitte*et al*., 2nd ed. 2003).

Slide agglutination test was used to serotype the isolates of *Salmonella* and *Shigella* species using polyvalent/monovalent antisera. Presumptive *Salmonella* colonies were confirmed by genus-specific PCR [18]. The slide agglutination test for serotyping of *Shigella* species was carried out using antisera following the manufacturer instruction (Remel Europe Ltd). The *Salmonella* isolates were serotyped at the Public Health Agency of Canada, National Microbiology Laboratory at Guelph, OIE Salmonella Reference Laboratory, Guelph, Ontario [19].

### Antimicrobial susceptibility

Antibacterial susceptibility test for the six *Shigella* and three *Salmonella* isolates was performed on Mueller-Hinton agar plates (SRL, India) using the Kirby-Bauer technique. The 0.5 McFarland standard used to prepare inoculum for the antimicrobial disk diffusion susceptibility test [20]. The antimicrobial susceptibility of bacterial isolates were screened for ten antibiotics amikacin (AK 30 μg), ampicillin (AM, 10 μg), amoxicillin (AX, 10 μg), cotrimoxazole (SXT, 25 μg), cefotaxime (CF, 30 μg), chloramphenicol (CH, 30 μg), ciprofloxacin (CP, 5 μg), gentamycin (GM, 10 μg), nalidixic acid (NA, 30 μg), and tetracycline (TTC, 30 μg).

The plates were incubated at 37 °C for 24 h, and the zone of inhibition diameters were measured with a ruler and, interpreted according to CLSI guidelines, and the results recorded as sensitive (S), resistant (R), or intermediate (I) based on CLSI [20]. The study declared multidrug resistance (MDR) if the isolates were resistant to more than two (> 2) of the antimicrobial agents belonging to different classes [21, 22]. *Escherichia coli* ATCC 25922 used as a quality control strain during the antimicrobial susceptibility test [20].

### Statistical analysis

The data were entered into EpiData 3.02 and then transferred to SPSS version 17.0 statistical software for data processing and analysis. The descriptive statistics such as mean, standard deviation, and proportion used.

## Results

A total of 239 children included in the study. Of these, more than three-quarters of them were from Ambo Hospital. Slightly more than half (52.3%) of the children were boys, and two-third of all the children were from urban areas. The children’s age ranged from 5 to 60 months, with a mean of 27.96 (SD ± 17.09) months (Tables 1, and 2).

**Table 1:** Socio-demographic characteristics of study participants attended Ambo public health facilities, 2014

Variables	Categories	Frequency	Percentage (%)
Health Institution	Awaro Health Center	24	10.0
	Ambo Health Center	34	14.2
	Ambo Hospital	181	75.7
Residence	Urban	162	67.8
	Rural	77	32.2
Gender	Male	125	52.3
	Female	114	47.7
Household workers	≥1	211	88.3
	None	28	11.7
Domestic animals in the house	Yes	97	40.6
	No	142	59.4
Source of drinking water	Tap water	170	71.1
	Domestic well water	13	5.4
	River water	42	17.6
	Public hand pump water	14	5.9
Type of diarrhea	Watery diarrhea	49	20.5
	Bloody diarrhea	32	13.4
	Mucoid diarrhea	100	41.8
	Loose stool	58	24.3
Household member/room	< 1.5 persons-per-room	198	82.8
	> 1.5 persons-per-room	41	17.2
Antibiotics prescribed in last 4 weeks	Cotrimoxazoale	10	4.2
	Ceftriaxone	2	0.8
	Amoxicillin	1	0.4
	Metronidazole	2	0.8

**Table 2:** Distribution of the *Salmonella* and *Shigella* among children who attended Ambo public health facilities, 2014

Age in Months	*Salmonella* species	*Shigella* species			Total
		SF	SB	SS	
0–6	0	0	0	0	0
7–12	1(0.4%)	0	0	0	1(0.4%)
13–24	1(0.4%)	2(0.8%)	1(0.4%)	0	4(1.7%)
25–36	1(0.4%)	0	0	1(0.4%)	2(0.8%)
37–48	0	1(0.4%)	0	0	1(0.4%)
49–60	0	0	1(0.4%)	0	1(0.4%)
Total	3(1.3%)	3(1.3%)	2(0.8%)	1(0.4%)	9(3.8%)
		6 (2.5%)			

Key: *Shigella flexneri (SF), Shigella boydii (SB), Shigella sonnei (SS*), and none (−)

As shown in **Table, four** out of five children were living in non-crowded housing conditions, which is < 1.5 persons-per-room [23]. One out of six households was using water from unsafe sources such as rivers and domestic wells. Two out of five households with diarrheic children had at least one domestic animal in their household (Table 1). However, no statistically significant difference was observed in the proportion of identified enteric bacterial pathogens across socio-demographics characters.

### Clinical characteristics

**Fig. 1:**
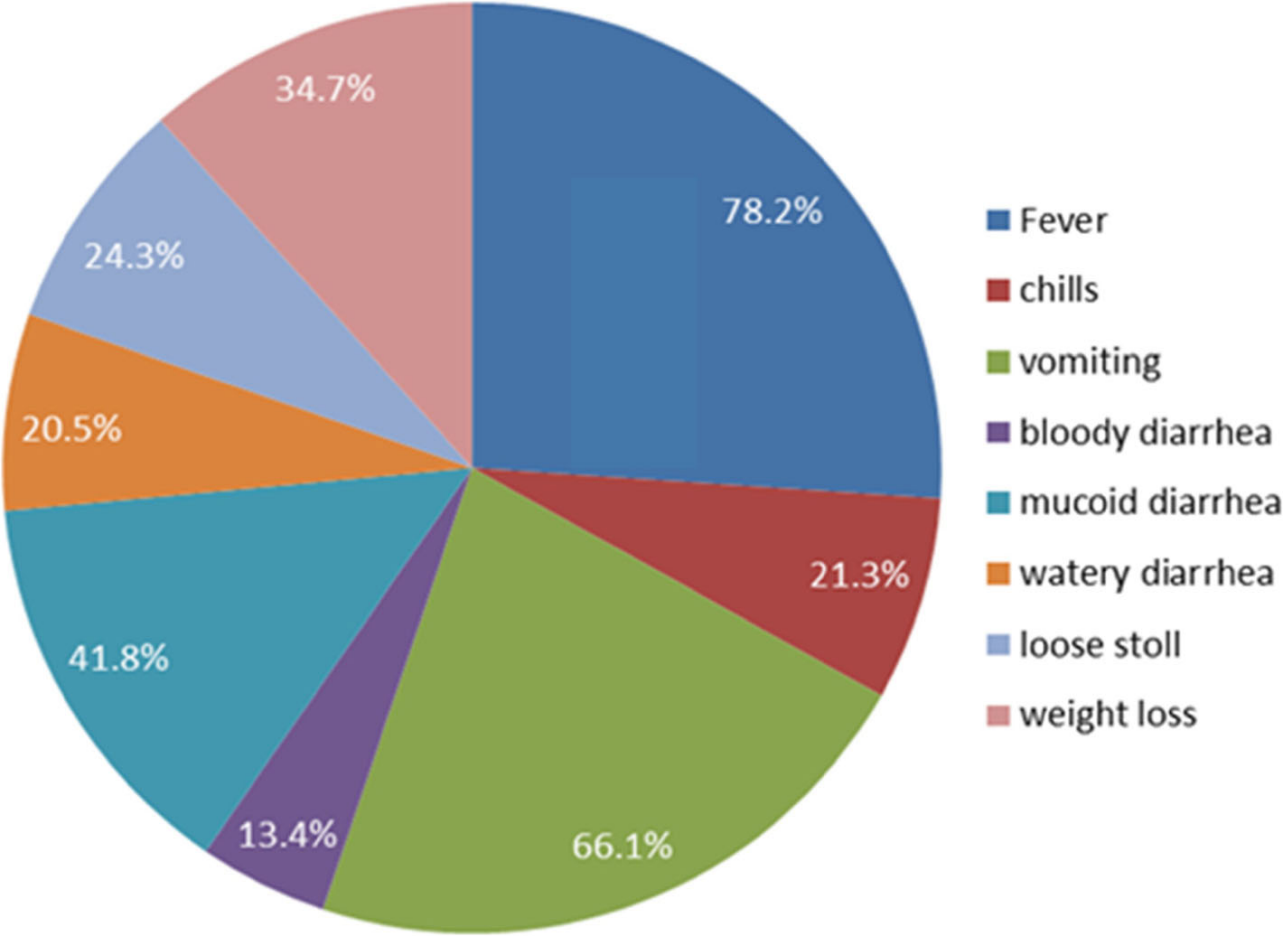
Clinical characteristics of diarrheic children attending Ambo public health facilities, 2014

Fifteen (6.3%) children took one of the four antibiotics: ceftriaxone, amoxicillin, metronidazole, or cotrimoxazole in the past four weeks—cotrimoxazole being the most commonly prescribed drug (Table 1). The most common clinical complaints recorded were fever (78.2%) followed by vomiting (66.1%), and mucoid diarrhea (41.8%) (Fig. 1).

**Fig. 2:**
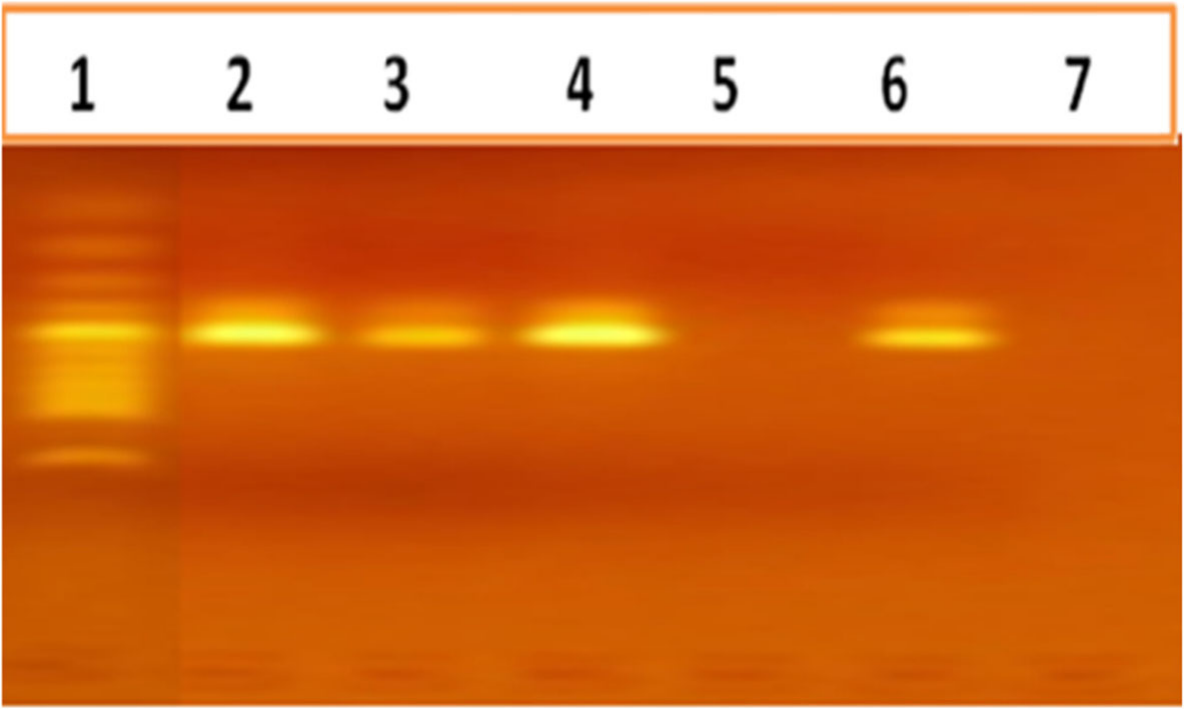
Ethidium bromide-stained 2% agarose gel showing the results of electrophoresis of products of the PCR reaction. A 496-bp band is seen in each lane with the product of the PCR for *Salmonella* species; bands are not seen in negative lanes. A 496-bp band is seen in lane 2, 3 and 4 with the product of the PCR for genus *Salmonella*. Lane 1 = ladder; Lane 2–5 = Clinical Isolate; Lane 6 = Positive control; Lane 7 = Negative control

From seven positive stool samples, six *Shigella* and three *Salmonella* species were isolated. Of the isolated *Shigella* three (1.3%) were *Shigella flexneri* (3; 1.3%), two (0.8%) were *Shigella boydii*, and one was (0.4%) *Shigella sonnei*. The three *Salmonella* (3; 1.3%) isolates were *S.* chicago*, S.* caracas, and *S*. saintpaul (Fig. 2, Table 3). From identified positive stool samples, while five had a single infection of bacterial pathogens, two were infected by both bacterial pathogens (Table 2).

**Table 3:** *Salmonella* Serotyping and Phage-Typing report from Public Health Agency of Canada, National Microbiology Laboratory at Guelph, OIE *Salmonella* Reference Laboratory, Guelph, Ontario at January 13, 2016 report

Submission	Isolation	Received	serotypes	Antigens
SA20160054	AW-gT	2015-10-19	S. chicago	28:r,[i]:1,5
SA20160055	77	2015-10-19	S. caracas	6,14,25,g,m,s”
SA20160056	89-T	2015-10-19	S. saintpal	4:e,h:1,2

Nineteen (7.9%) children were positive for the intestinal parasite. The most common identified intestinal protozoan was *Giardia lamblia* eight (3.3%), followed by *Entamoeba* cyst/trophozoite in five (2.1%) (Table 4). Fecal *leukocytes* and *RBC* were observed in 113 (55.6%) and 55 (23%) children, respectively. Despite the low proportion, mucoid diarrhea (2.1%) had a higher frequency of *Salmonella* and *Shigella* isolates (Table 5).

**Table 4:** Intestinal parasites identified among diarrheic children attending Ambo public health facilities, 2014

Age	Intestinal parasites (n, %)					
In Month	*Giardia lamblia*	*Entamoebaistolyticacyst/trophozoite*	*Ascarislumbricoides*	*Hook worm ova*	*Haymenolepis nana*	Total
0–6	0	0	0	0	0	0
7–12	0	0	0	0	0	0
13–24	0	0	0	0	0	0
25–36	1(0.4)	0	1(0.4)	0	0	2(0.8)
37–48	4(1.7)	0	1(0.4)	0	0	5(2.1)
49–60	3(1.3)	5(2.1)	2(0.8)	1(0.4)	1(0.4)	12(5.0)
Total	8(3.3)	5(2.1)	4(1.7)	1(0.4)	1(0.4)	19 (7.8)

**Table 5:** Types of diarrhea among children positive for *Salmonella* and *Shigella s*pecies infection attending Ambo public health facilities, 2014

Types of diarrhea	Types of isolate (n, %)				Total (n, %)
	*Salmonella* spp.	*Shigella* spp.			
		SF	SB	SS	
Watery diarrhea	1(0.4)	1(0.4)	0	0	2(0.8)
Bloody diarrhea	0	1(0.4)	1(0.4)	0	2(0.8)
Mucoid diarrhea	2(0.8)	1(0.4)	1(0.4)	1(0.4)	5(2.1)
Loose stool	0	0	0	0	0
Total	3 (1.2)	6 (2.5)			9 (3.8)

Key: *Shigella flexneri* (SF), *Shigella boydii* (SB), *Shigella sonnei* (SS) and (−) none

### Antimicrobial susceptibility of isolated *Salmonella* and *Shigella* isolates

The antimicrobial profile for isolated *Salmonella* and *Shigella* species (*n* = 9) to 10 antimicrobials drugs determined by the disk diffusion method following the recommendations of the CLSI [20]. The isolated *Salmonella* and *Shigella* species had variable degrees of resistance to all antimicrobial agents tested. The susceptibility of 31.1% of the isolates was below standard resistance breakpoints for ampicillin, amoxicillin, cotrimoxazole, chloramphenicol, nalidixic acid*, tetracycline,* and cefotaxime. The highest enteropathogenic bacteria resistance was observed against ampicillin (88.9%) followed by tetracycline (55.6%) and cotrimoxazole (55.6%), chloramphenicol (44.4%), amoxicillin (33.3%), nalidixic acid (11.1%), and cefotaxime (11.1%). All isolates were sensitive to amikacin, ciprofloxacin, and gentamycin.

The resistance against tested ten antimicrobials was higher for *Salmonella* species (33.3%) than the *Shigella* species (30%). The highest rate of resistance observed was against ampicillin, which was 100% for *Salmonella* species and 83.3% for *Shigella* species. Conversely, the least antimicrobial resistance was to amikacin, ciprofloxacin, and gentamycin, where all isolates were sensitive*. Shigella* species showed no to a low level of resistance for cefotaxime and nalidixic acid (16.7%), respectively. *Salmonella* species showed no resistance against nalidixic acid. The resistance against the most commonly prescribed antibiotics, cotrimoxazole was 66.7% for *Salmonella* and 50% for *Shigella* isolates (Table 6).

**Table 6:** Antimicrobial Susceptibility of isolated *Salmonella and Shigella* species among diarrheal children attending Ambo public health facilities, 2014

*Salmonella* and *Shigella* (n = 9)	Antibiotics types in percentage (%)										%	Total No. (%)
	AK-30	A −10	AML-2	SXT −25	CTX-5	C-30	CIP-5	GM-10	N A	Te-30		
*S. chicago*	S	R	I	R	S	R	S	S	S	R	40	33.3
*S. caracas*	S	R	S	R	R	R	S	S	S	R	50	
*S. saintpaul*	S	R	S	S	S	S	S	S	S	S	10	
*Shigella flexneri*	S	R	R	I	S	S	I	S	S	R	30	30
*Shigella flexneri*	S	R	I	I	S	I	S	S	S	S	10	
*Shigella flexneri*	S	R	I	R	S	S	S	S	S	S	20	
*Shigella sonnei*	S	I	R	R	S	S	S	S	S	R	30	
*Shigella boydii*	S	R	I	R	S	R	S	S	R	R	50	
*Shigella boydii*	S	R	R	S	S	R	S	S	S	R	40	
Total resistance (%)	0	88.9	33.3	55.6	11.1	44.4	0	0	11.1	55.6	31.1	

Key: Amikacin (AK-30 μg), Ampicillin (A-10 μg), Amoxicillin (AML-10 μg), Cotrimoxazole (SXT −25 μg), Cefotaxime (CTX-30 μg), Chloramphenicol (C-30 μg), Ciprofloxacin (CIP-5 μg), Gentamycin (GM-10 μg), Nalidixic acid (NA-30 μg), Tetracycline (Te-30 μg), sensitive (S), resistance (R) and intermediate (I)

Regarding multidrug resistance, five *Shigella* species and two *Salmonella* species were multidrug-resistant. Out of the resistant species while, two (22.2%) were resistant to one antimicrobial two (22.2%) were resistant to five antibiotic agents (Table 6).

## Discussion

This study intended to examine the prevalence, and antimicrobial susceptibility status of *Salmonella* and *Shigella* strains isolated from under-five children with diarrhea presented to Ambo town public health institutions. The overall prevalence of *Salmonella* and *Shigella*infection was 3.8%, and there was a high rate of multidrug resistance, especially for *Salmonella* species. While the highest resistance observed against ampicillin, the least was for amikacin, ciprofloxacin, and gentamycin.

The overall prevalence of *Salmonella* and *Shigella* infection was less than the findings from Jimma, in Southwestern Ethiopia (8.4%) [7], Southern Ethiopia (22.5%) [14], and rural coastal India (11.2%) [24]. On the other hand, the magnitude of isolated *Shigella* species *(*2.5%) was comparable to findings from Jimma, Southwest Ethiopia (2.3%) [7], Nekemte (2.1%) [25], and Addis Ababa (3.2%) [13]. Conversely, the *Shigella* isolation rate was lower than the results from Jimma (8.8%) [13], Butajira, Central Ethiopia (4.5%) [9], and rural coastal India (4.2%) [24].

The lower S*higella* and *Salmonella* isolation might be due in part to other potential enteric pathogen causes of diarrhea such as *Rotavirus, Campylobacter* species, *Yersinia enterocolitica, Aeromonas* species, and *protozoans*.

Among *Shigella* isolates, *Shigella flexneri* (12.5%) was the most dominant, followed by *Shigella boydii* (8.3%) and *Shigella sonnei* (1.4%), which is comparable with a study done in Northern India [26]. On the contrary, study findings in Jimma, Southwest Ethiopia, and Salvador, Bahia, Brazil showed that the isolates were not comparable [8, 27], respectively. The discrepancy might be due to a difference in the study population, study time, and illness due to other enteric pathogens.

The rate of identification of *Salmonella* species(1.3%) in this study was comparable to other studies done in Ethiopia, 1% from Hossana [28] and elsewhere 1.6% from North a [26]. However, it was lower than other studies in Ethiopia 7.8% from Bahir Dar town [12], but lower than a study done in, Ethiopia (6.2% from Jimma, [7], and other studies focused on multidrug-resistant *Salmonella concord* among children in Jimma (2.5%) and Addis Ababa (6.7%) [13], Hawassa (2.5%) [14], and Butajira(10.5%) [9]. The reason might be the difference in susceptibility methods breakpoints used.

There was a high rate of resistance to ampicillin for both *Salmonella* and *Shigella* species. Ampicillin is among the top dispensed drugs in Ethiopia for the last several years. The majority of such antibiotics (85%) are prescribed empirically [29]. That has made ampicillin familiar, popular, and accessible among the people, hence leading to a high rate of self-prescription. According to Mihrate et al., (2014), Ampicillin (11.1%) is the third most common self-prescribed drug only next to amoxicillin (61.1%) and, cotrimoxazole (27.8%). The self-prescription such antibiotics are common for gastrointestinal disorders [30, 31]. Similarly, high levels of resistance were reported in previous studies [32–34]. The public health implication of such high resistance could be associated with the suboptimal water and sanitation conditions and inadequate sewage disposal systems. This could be further complicated by the hand hygiene practice of caregivers and/or mothers.

All *Salmonella* and *Shigella* isolates in this study displayed resistance to one or more antimicrobial, including ampicillin, tetracycline, amoxicillin, cotrimoxazole, nalidixic acid, and cefotaxime. Among the isolates, there were no resistances for amikacin, ciprofloxacin and, gentamycin—except 1 and 2 intermediate for ciprofloxacin and gentamycin, respectively, which is comparable with other studies [11, 14, 24, 27, 35]. The highest antibiotic resistance of *Shigella* against ampicillin (83.5%) observed was comparable with a study done on *Shigella* isolates in Awassa (93%) [10], Jimma (70.1%) [8], Gondar (79.9%) [36], Harar (100%) [11], Jimma (100%) [7], and Southwestern Nigeria (90.5%) [37]. However, it is higher than a study done in Hawassa (63.6%) [14] and Butajira (47.1%) [9]. The differences may be due to the different susceptibility methods breakpoints used [38].

The antibiotic resistance of *Shigella* species to tetracycline (66.7%) was comparable with a study done in Jimma (63.6%) [8] and in Harar (70.6%) [11], but lower than a study done in other parts of Ethiopia; in Butajira (82.4%) [9], in Gondar University teaching hospital (86%) [39], in Awassa (90%) [10], and Gondar (86%) [36]. The difference might be due to strains that are moderately susceptible to tetracycline in some areas of the country. Cotrimoxazole showed 50% resistance against *Shigella* which is comparable with a study done in Awassa (56.0%) [10] and Addis Ababa (45.7%), [40]. Fifty percent of *Shigella* spp. showed resistance to amoxicillin, which was not comparable with studies done in Hawassa [14], Harar [11], Jimma [7] which count 100%, and Southwestern Nigeria which counts 81% [37]. The difference might be attributed to the difference in laboratory techniques used for the susceptibility test [38].

Antibiotic resistance against chloramphenicol 33.3% in this study is comparable with a study done in Harar (29.5%) [11], in Butajira (29.4%) [9] and Egypt [41], but was lower than a study done in Gondar University Hospital, Northwest Ethiopia [36], Awassa (63.3%) [10], and Jimma (40.3%) [8]. This result is also not comparable to a study done in Southwestern Nigeria (85.7%) [37]. The differences in the findings might be due to a biophysical environment and antibiotic resistance [42].

The study suggested that resistance shadowed a ‘selection density’. In the assumption, ecology was the basis; i.e., in a particular geographic area as the more antibiotic used for individual persons, animals, or plants, there is a high probability of bacteria to develop antibiotic resistance. Antibiotic residues from human excreta stool can have an impact on the geographical ecosystem. They can easily pass to water, and soil thought manure and sewage. So, antibiotic-resistant bacterial strains may have been found in natural water [43].

This study has a small sample size and included only children with diarrhea and restricted to a public health facility, to this end, it will be difficult to generalize beyond the study settings. Besides, restricting the finding to *Shigella* and *Salmonella* species hindered the isolation of other common causes of diarrhea. Thus, a comprehensive study should be conducted to determine the common enteric pathogens that cause diarrhea along with sensitivity tests.

## Conclusion

The low prevalence of *Salmonella* and *Shigella* species was identified from the diarrhea stool of children under five years in the study area. Among those isolates, antibiotic resistance was at a high rate against ampicillin. It was followed by cotrimoxazole and tetracycline while the isolates were sensitive to amikacin, ciprofloxacin, and gentamycin.

## Data Availability

The datasets used and analyzed in the study are available from the corresponding author on reasonable request.

## Abbreviations

ATCC: American Type Culture Collection
ATPHI: Ambo Town Public Health Institutions
AUML: Ambo University Microbiology Laboratory
CLSI: Clinical and Laboratory Standards Institute
CSA: Central Statistical Agency
EBR: Ethiopian Birr
EPEC: Enteropathogenic *Escherichia coli*
EPHI: Ethiopian Public Health Institution
OPD: Outpatient Departments
Spp.: Species
SPS: Statistical Package for Social Science
SS: *Salmonella Shigella* Medium
USA: United States of America
WHO: World Health Organization
XLD: Xylose lysine desoxycholate agar
